# New Porous Heterostructures Based on Organo-Modified Graphene Oxide for CO_2_ Capture

**DOI:** 10.3389/fchem.2020.564838

**Published:** 2020-09-17

**Authors:** Eleni Thomou, Evmorfia K. Diamanti, Apostolos Enotiadis, Konstantinos Spyrou, Efstratia Mitsari, Lamprini G. Boutsika, Andreas Sapalidis, Estela Moretón Alfonsín, Oreste De Luca, Dimitrios Gournis, Petra Rudolf

**Affiliations:** ^1^Department of Materials Science and Engineering, University of Ioannina, Ioannina, Greece; ^2^Zernike Institute for Advanced Materials, Faculty of Science and Engineering, University of Groningen, Groningen, Netherlands; ^3^National Center for Scientific Research “Demokritos”, Athens, Greece

**Keywords:** organosilica, graphene oxide, pillaring, hybrid structures, porous heterostructures, sorbents, CO_2_ capture

## Abstract

In this work, we report on a facile and rapid synthetic procedure to create highly porous heterostructures with tailored properties through the silylation of organically modified graphene oxide. Three silica precursors with various structural characteristics (comprising alkyl or phenyl groups) were employed to create high-yield silica networks as pillars between the organo-modified graphene oxide layers. The removal of organic molecules through the thermal decomposition generates porous heterostructures with very high surface areas (≥ 500 m^2^/g), which are very attractive for potential use in diverse applications such as catalysis, adsorption and as fillers in polymer nanocomposites. The final hybrid products were characterized by X-ray diffraction, Fourier transform infrared and X-ray photoelectron spectroscopies, thermogravimetric analysis, scanning electron microscopy and porosity measurements. As proof of principle, the porous heterostructure with the maximum surface area was chosen for investigating its CO_2_ adsorption properties.

## Introduction

The dramatic effects of global warming (sea level rise, wildfires, flooding, extreme weather conditions) and the constant degradation of our planet are among the most important and challenging issues the modern world is facing. Approximately 35 billion metric tons of CO_2_ are emitted globally each year (National Academies of Sciences Medicine, 2019) and with the already existing technology only an insignificant fraction of these emissions is currently prevented from contributing to the greenhouse gas effect. Instead of being considered a detrimental waste product, carbon dioxide can become a precious and perfectly sustainable source material, which, once captured, can be utilized in a plethora of commercial processes such as enhanced oil recovery, chemical or biological conversion, food industry, mineral carbonation *etc*. (National Academies of Sciences Medicine, 2019). Therefore the development of low cost, efficient, easily applied, reusable and environmentally friendly materials capable of capturing and storing greenhouse gases, and more specifically CO_2_, which constitutes a major factor in global warming, is more urgent than ever before.

Adsorption that involves binding of carbon dioxide onto the surface of a solid sorbent, is one of the most promising CO_2_ separation technologies that are currently being developed and used (Leung et al., 2014). A wide range of sorbents has been proposed, from activated carbon, to zeolites, alumina, or MOFs—just to name a few (Shi et al., 2020) and among the materials that have been studied for this purpose is also graphene oxide (dos Santos and Ronconi, 2017; Huang and Feng, 2018; Pokhrel et al., 2018; Shrivastava et al., 2018). Graphene-based materials hold a prominent position as they combine a series of significant advances such as excellent physicochemical properties, high specific surface areas, low adsorption energy, high selectivity and light weight (Novoselov et al., 2012). Graphene oxide (GO), a layered structure produced by the treatment of graphite with strong oxidizing agents and characterized by a high concentration of diverse oxygen-containing functional groups, which make it hydrophilic, has been identified as an excellent host matrix for diverse functional molecular structures. Organic molecules, inorganic pillars or metal ions can be accommodated in its interlayer space to design porous hybrid materials for energy (Enotiadis et al., 2012), environmental (Duan et al., 2017) and sorption (Pedrielli et al., 2018) applications. An essential step for realizing such nanostructures was the modification/intercalation of graphene oxide with primary aliphatic chains in order to create an organophilic GO derivative that can be readily dispersed in polar organic solvents (Bourlinos et al., 2003).

Large specific surface area and high selectivity are two of the most important characteristics a material should have in order to be considered a suitable candidate for CO_2_ adsorption applications (Leung et al., 2014). Saha and Kienbaum showed that higher selectivity for CO_2_ over other gases can be achieved by introducing selected functionalities and heteroatoms (nitrogen, oxygen, sulfur) to the sorbent's surface (Saha and Kienbaum, 2019). However, it is quite challenging to meet the high specific surface area criteria when employing GO for the development of sorbents since the big disadvantage of layered materials like GO is their lack of permanent porosity: a normal layered material can for example swell upon hydration, but collapses again after dehydration. Hence, in order to overcome this obstacle, taking advantage of intercalation chemistry, permanent pillars have to be introduced between the layers in order to create a robust 3-D network of adjacent graphene sheets with nanopores of the right size and surface properties to accommodate CO_2_.

Pillaring of 2-D layered materials allows for a fine control of the structural characteristics of the resulting micro- and nanoporous composites (Ohtsuka, 1997) and assures structural stability, permanent pore sizes and high surface areas. Such rational interlayer design has opened new prospects for applications in areas as diverse as the nanocomposites themselves (Nicotera et al., 2011; Enotiadis et al., 2013; Zapata et al., 2013), namely catalysis (Kloprogge et al., 2005; Gil et al., 2008), metal uptake (Balomenou et al., 2008), sensors (Tonlé et al., 2007), environmental remediation (Zhao et al., 2011), supercapacitors (Yan et al., 2012; Ke and Wang, 2016; Banda et al., 2019) and Lithium-ion batteries (Hu et al., 2019).

In cases where an improved structural stability was crucial, graphene oxide sheets were kept apart *via* the creation of metal-oxide networks in the interlayer space (Matsuo et al., 2005). Many studies have been published regarding the pillaring of graphene-based materials based on the incorporation of different silicon sources mainly by the sol-gel method. Organosilanes are silicon sources that have already been used successfully for the synthesis of periodic mesoporous organosilicas (PMOs) in the form of thin films (Wahab and He, 2009; Wahab et al., 2009) and fibers (Wahab et al., 2006), as well as materials with other morphologies depending on the choice of precursor and the experimental conditions (Wahab et al., 2004a,b, 2005). Matsuo's group has studied extensively the synthetic conditions for pillared GO with various silylating reagents such as 3-aminopropylethoxysilanes or alkyl trichlorosilane with various alkyl lengths (Matsuo et al., 2007, 2009). A pyrolysis step is required for these hybrids to obtain large surface areas and controlled pore sizes, both essential characteristics to extend their use to applications in the fields of hydrogen storage (Matsuo et al., 2012b), catalysts (Maruyama et al., 2014; Rana et al., 2015), electrodes (Yoo et al., 2011) and sensors (Duan et al., 2017).

Matsuo et al. have shown that the BET surface area of porous graphene heterostructures can be increased up to 756 m^2^/g after insertion of two different organosilanes between graphene layers in a two-step process (Matsuo et al., 2012a). The same group also showed that repeating silylation process of graphene oxide with trichlorosilane affects the density of the siliceous pillars, and allows to tailor the BET surface area between 77 and 723 m^2^/g (Matsuo et al., 2015).

In this work, a new effective and efficient silylation process is proposed for the development of high surface area materials, which is considerably faster than the ones reported in the literature so far (Matsuo et al., 2012a; Maruyama et al., 2014). The silylation method was performed on an organically modified GO derivative employing three distinct silica precursors with different structural characteristics and chosen to develop silica networks within the interlayer space of the organo-modified GO. The final products were fully characterized with a combination of techniques and the degree of silylation of each reagent was evaluated. The one bridged with a phenyl group showed the maximum amount of silica content in the final heterostructure. Subsequent pyrolysis was found to create the desired porous structure raising the samples' specific surface area up to 550 m^2^/g. For this hybrid the potential as sorbent of CO_2_ was briefly explored.

## Experimental Section

### Materials

Graphite (purum, powder ≤ 0.2 mm), Nitric acid (65 % HNO_3_) and Potassium chlorate (KClO_3_, 98+%) were purchased from Fluka Inc. Dodecylamine (DA, ≥ 99%), 1,4-bis(triethoxysilyl)-benzene (BTB 99%), tetraethylorthosilicate (TEOS 98+%) and (3-aminopropyl)triethoxysilane (APTEOS ≥ 98%) were acquired from Sigma-Aldrich, and sulfuric acid (H_2_SO_4_, 95–97%), n-butanol and sodium hydroxide (NaOH) were obtained from Merck. All reagents were of analytical grade and used without further purification. Distilled deionized water was used for all the experiments.

### Graphene Oxide Synthesis

Graphene oxide, denoted as GO in the following, was synthesized using a modified Staudenmaier's method (Staudenmaier, 1898; Gengler et al., 2010; Stergiou et al., 2010). In a typical synthesis, 10 g of powdered graphite was added to a mixture of 400 mL of 95–97% H_2_SO_4_ and 200 mL of 65% HNO_3_, while cooling in an ice–water bath in order to counteract the heat released during the very exothermic chemical reaction. 200 g of powdered KClO_3_ was added to the mixture in small portions under continuous stirring and cooling. The reaction was quenched after 18 h by pouring the mixture into distilled water and the oxidation product was washed until the pH reached an almost neutral value (~6.0), and finally air-dried at room temperature after being spread on a glass plate.

### Organo-Modified Graphene Oxide

1.5 g of dodecylamine were dissolved in 50 mL of ethanol and the solution was added slowly to an aqueous GO suspension (beforehand 0.1 M NaOH was added to adjust the pH value to ~7.5) under vigorous stirring (dodecylamine/GO 3:1 w/w). The mixture was stirred for 24 h, centrifuged, washed three times with ethanol/ water: 1/1, and air-dried after being spread on a glass plate. The organo-modified GO is denoted as org-GO.

### Silica-GO Heterostructures

**Figure 1:**
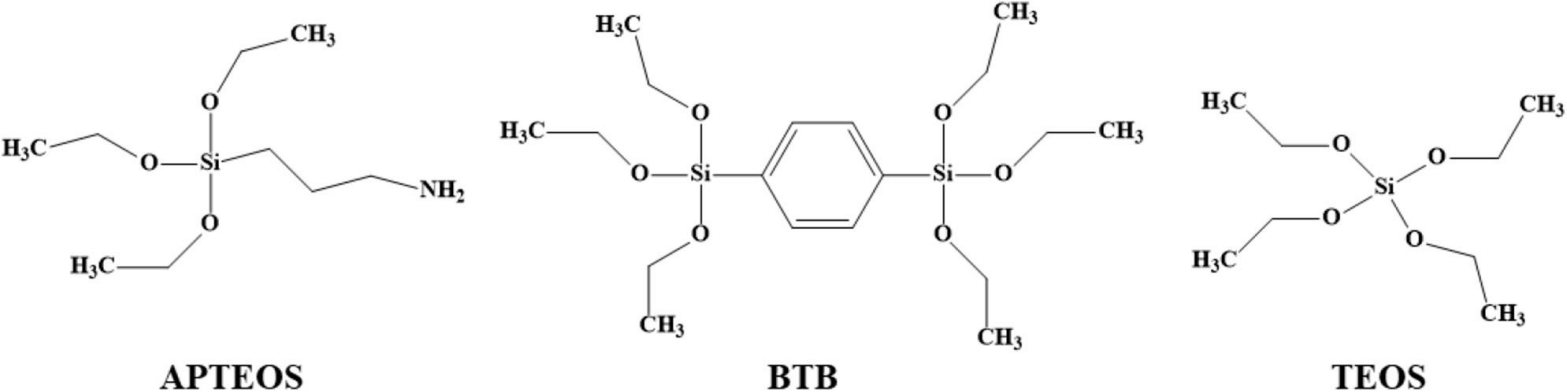
Chemical structures of the silica precursors used (APTEOS, BTB, and TEOS).

Before silylation, GO was dried a second time under vacuum at room temperature overnight. 100 mg of org-GO were dispersed in n-butanol (5 mL), sonicated for 30 min and left under stirring overnight. The silica precursor (APTEOS, BTB or TEOS; chemical structures shown in Figure 1) was slowly added to the org-GO dispersion under stirring for 2 h before adding the water (containing a drop of hydrochloric acid) while keeping constant the molar ratio silica precursor/H_2_O/n-butanol: 1/4/54. The sol-gel reactions were performed at 50°C while stirring, and the obtained gel was placed in the oven at 50°C overnight. The silica–GO organo-heterostructures are denoted as GO-BTB, GO-APTEOS and GO-TEOS, depending on which of the three organosilica precursors was employed. The final porous structures were collected after calcination in air at 370°C for 120 min. The calcinated samples are denoted as G-BTB, G-APTEOS, and G-TEOS.

### Characterization Techniques

The powder X-ray diffraction (XRD) patterns were collected on a D8 Advanced Bruker diffractometer with a Cu K_α_ X-ray source (40 kV, 40 mA) and a secondary beam graphite monochromator. The patterns were recorded in a 2θ range from 2 to 40°, in steps of 0.02° and with a counting time of 2 s per step. Fourier transform infrared (FTIR) spectra in the range 400–4000 cm^−1^ were measured with a Shimadzu FT-IR 8400 spectrometer equipped with a deuterated triglycine sulfate (DTGS) detector. Each spectrum was the average of 64 scans collected at 2 cm^−1^ resolution. Samples were in the form of KBr pellets containing ca. 2 wt% sample. X-ray Photoelectron Spectroscopy (XPS) analysis was performed using a Surface Science SSX-100 ESCA instrument with a monochromatic Al Kα X-ray source (hν = 1486.6 eV). The pressure in the measurement chamber was maintained at 1 × 10^−9^ mbar during data acquisition. The electron take-off angle with respect to the surface normal was 37°. The XPS data were acquired by using a spot size of 1,000 μm in diameter and the energy resolution was 1.3 eV for both the survey spectra and the detailed spectra of the C1*s*, O1*s*, and Si2*p* core level regions. Binding energies are reported ±0.1 eV and referenced to the C1*s* photoemission peak centered at a binding energy of 284.8 eV (Moulder et al., 1995). All XPS spectra were analyzed using the least-squares curve-fitting program Winspec (developed at LISE laboratory of the University of Namur, Belgium). Deconvolution of the spectra included a Shirley baseline (Shirley, 1972) subtraction and fitting with a minimum number of peaks consistent with the chemical structure of the sample, taking into account the experimental resolution. The profile of the peaks was taken as a convolution of Gaussian and Lorentzian functions. The uncertainty in the peak intensity determination is 2 % for all core levels reported. For the measurements, evaporated polycrystalline gold films supported on mica (grade V-1, TED PELLA), prepared by sublimation of 99.99 % gold (Schöne Edelmetaal B.V.) as detailed in Mendoza et al. (2007) were used as substrates. Thermogravimetric (TGA) and differential thermal (DTA) analyses were performed using a Perkin Elmer Pyris Diamond TG/DTA. Samples of approximately 5 mg were heated in air from 25 to 850 °C, at a rate of 5° C/min. The nitrogen adsorption-desorption isotherms were measured at 77 K on a Sorptomatic 1990 Thermo Finnigan porosimeter. Surface area values were determined by the Brunauer–Emmett–Teller (BET) method. The surface morphology of the materials was studied using a JEOL JSM-7401F field emission scanning electron microscope (FE-SEM). A low acceleration voltage was applied (~2 kV), and the working distance was set to 3 mm. A powder sample was mounted onto the round brass substrate using double-coated conductive carbon tape. CO_2_ adsorption isotherms at 0, 10, and 20 °C were measured on an Intelligent Gravimetric Analyser (IGA–Hiden Ltd.). Before exposure to CO_2_ the samples were outgassed overnight in 250 °C under high vacuum (10^−8^ mbar) until the mass was observed to remain constant.

## Results and Discussion

**Figure 2:**
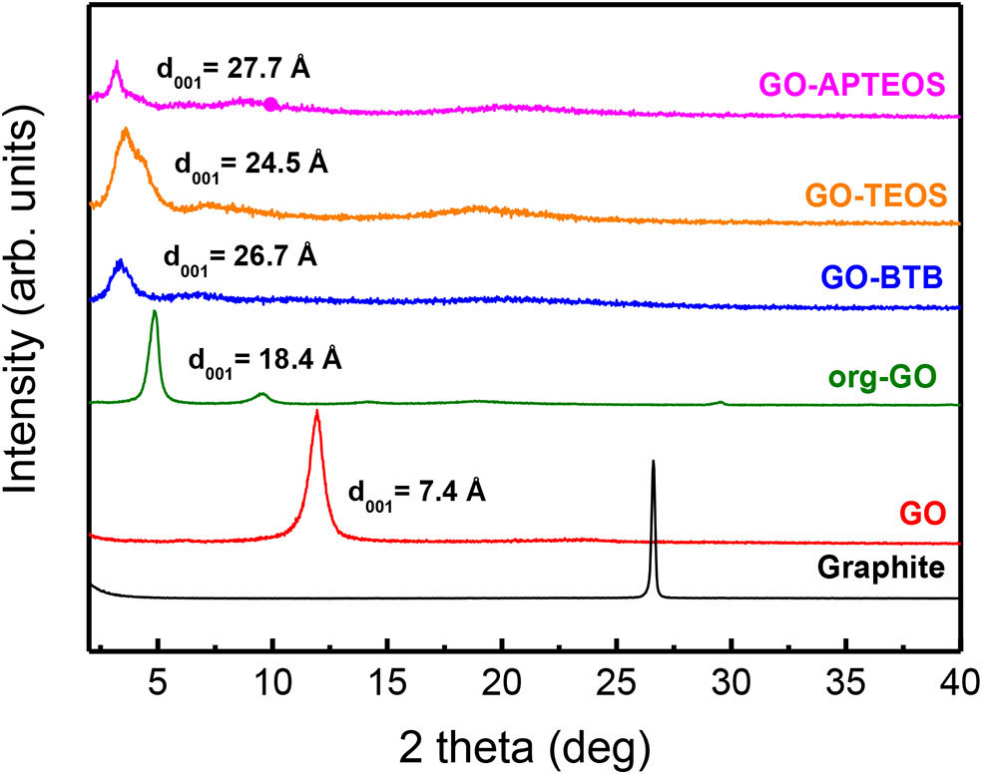
XRD patterns of pristine graphite, graphene oxide, organo-modified GO (org-GO), and heterostructures prepared by silylation with the three different organosilane precursors: BTB, TEOS, and APTEOS (GO-BTB, GO-TEOS, GO-APTEOS).

The XRD patterns of the parent materials and of the final hybrids after the sol-gel modification are shown in Figure 2. The XRD pattern of pristine graphite shows the characteristic peak corresponding to the 002 reflection of graphite at 26.6°, which translates into a basal spacing of 3.3 Å. This peak disappeared after the oxidation process, when the 001 reflection peak is found at 12.0°, corresponding to a basal spacing of 7.3 Å, characteristic of layered GO (Enotiadis et al., 2012). In fact, due to its hydrophilicity, GO exhibits one-dimensional swelling and can exhibit basal spacings between 6.1 and 11 Å, depending on the amount of water adsorbed (Dekany et al., 1998). After exposure of GO to dodecylamine the 001 reflection moved to even lower angles, attesting to an increase of the d_001_ spacing of GO and hence to the successful insertion of the guest molecules in the interlayer galleries. More specifically, the basal spacing, d_001_, of the org-GO shifts to 18.4 Å corresponding to an interlayer separation of Δ= 18.4–6.1 = 12.3 Å, where 6.1 Å is the thickness of the GO monolayer (Dekany et al., 1998). Through the organo-modification, the interlayer space of the GO becomes more accessible and readily modifiable with the silica precursors.

After the sol-gel reaction with silica alkoxides, the 001 reflection of the final hybrid materials is shifted to lower angles than for org-GO, confirming the successful expansion of the interlayer space and suggesting the formation of a silica network for each precursor. When org-GO was treated with the TEOS (GO-TEOS), the d spacing was calculated to amount to ~24.5 Å and even larger d values (26–27 Å) were achieved for APTEOS and BTB. The diffraction peak in this case is broader as a result of a distribution of different conformations. These larger values may be attributed to steric effects from the amino-terminated alkyl chains or phenylene ring anchored/bridged by silane centers of APTEOS and BTB, respectively.

**Figure 3:**
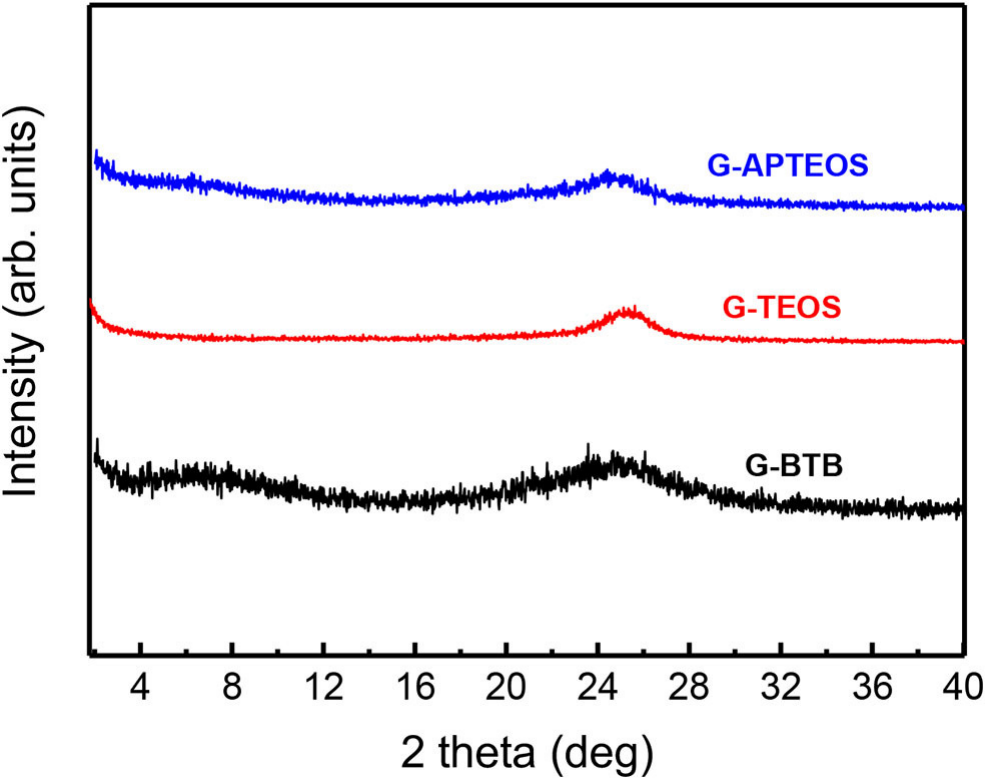
XRD patterns of the heterostructures obtained after calcination of GO-APTEOS, GO-TEOS and GO-BTB at 370°C to yield G-APTEOS, G-TEOS and G-BTB.

Figure 3 displays the XRD patterns of the silica-GO heterostructures obtained after calcination of GO-BTB, GO-TEOS and GO-APTEOS at 370 °C. For all hybrid heterostructures, the 001 reflection at lower angles (2–10°) is not clearly identifiable but very broad features are observed. This indicates that the graphene layers have lost their ability to stack and proves that the silica-GO heterostructures are in an exfoliated form due to the violent reduction of graphene oxide upon heating (Zhang et al., 2013).

**Figure 4:**
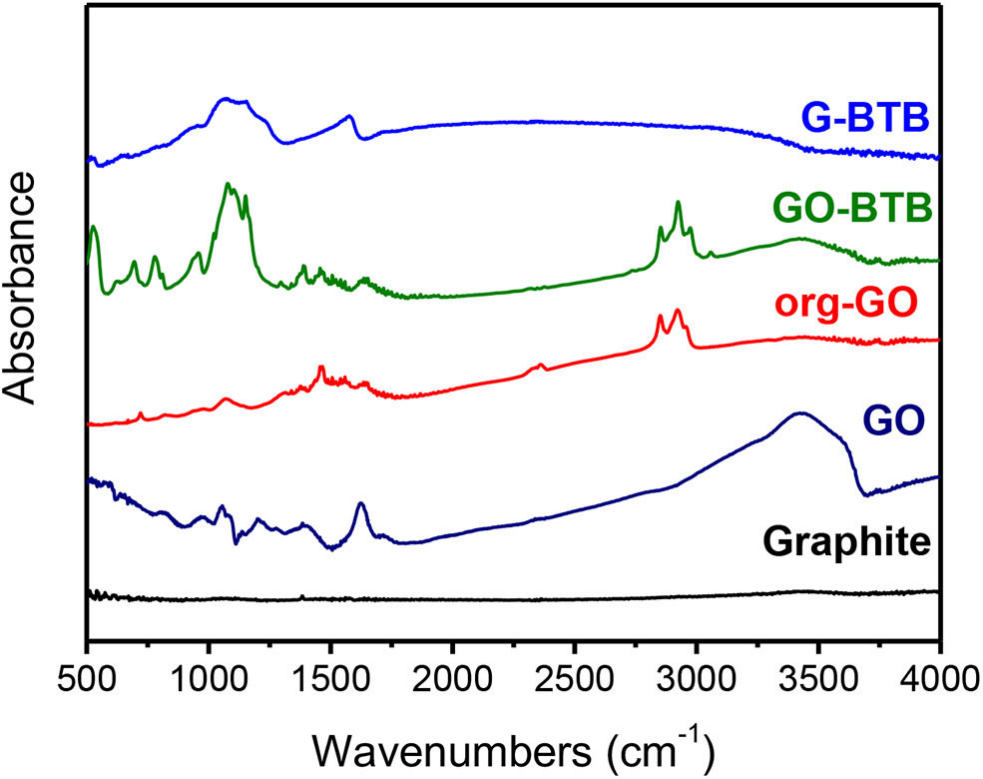
FTIR spectra of the heterostructure prepared by silylation of organo-modified graphene oxide with 1,4-Bis(triethoxysilyl)-benzene before (GO-BTB) and after (G-BTB) calcination; the spectra of graphite, pristine graphene oxide (GO) and graphene oxide intercalated with dodecylamine (org-GO) are plotted for comparison.

FTIR and XPS spectroscopies can provide additional information on the elemental composition and the type of chemical bonds present in the final hybrid heterostructures. Figure 4 compares the FTIR spectra of GO-BTB before and after (G-BTB) the calcination treatment with those of graphite, pristine (GO) and organo-modified graphene oxide (org-GO). Compared to that of GO, the spectrum of org-GO shows two additional bands at 2847 and 2919 cm^−1^ associated with the asymmetric and symmetric stretching vibrations of CH_2_ groups as well as a band at 1450 cm^−1^ due to the vibrations of the N-H bond of the amino group molecules. These spectral signatures clearly serve as evidence for the presence of dodecylamine in org-GO and thereby testify to the success of the organo-modification.

The spectra of the heterostructure obtained by reaction with the silica precursor BTB exhibit a set of new peaks before (GO-BTB) and after calcination (G-BTB), which can be attributed to vibrations of the silicate matrix: the peaks at 520 cm^−1^, 1065 and 1151 cm^−1^ are due to Si-O-Si vibrations, while the ones at 690 cm^−1^ and 950 cm^−1^ are assigned to the vibrations of O-Si-O. Similar spectral signatures were also found for the other heterostructures (GO-APTEOS, GO-TEOS, and G-APTEOS, G-TEOS, see supporting information, Figure S1) confirming the presence of silica networks in all three heterostructures before and after calcination. For the BTB precursor, two new peaks at 1396 and 3059 cm^−1^ corresponding to the vibrations of the double bond C=C and C-H of the phenyl rings appear in the GO-BTB spectrum. The characteristic bands of the intercalated dodecylamine identified in the spectrum of org-GO (2847, 2919 and 1483 cm^−1^) are absent from the spectrum of G-BTB, pointing to the successful removal of the surfactant molecules and hence give a first hint that porous silica-GO heterostructures developed during calcination.

**Figure 5:**
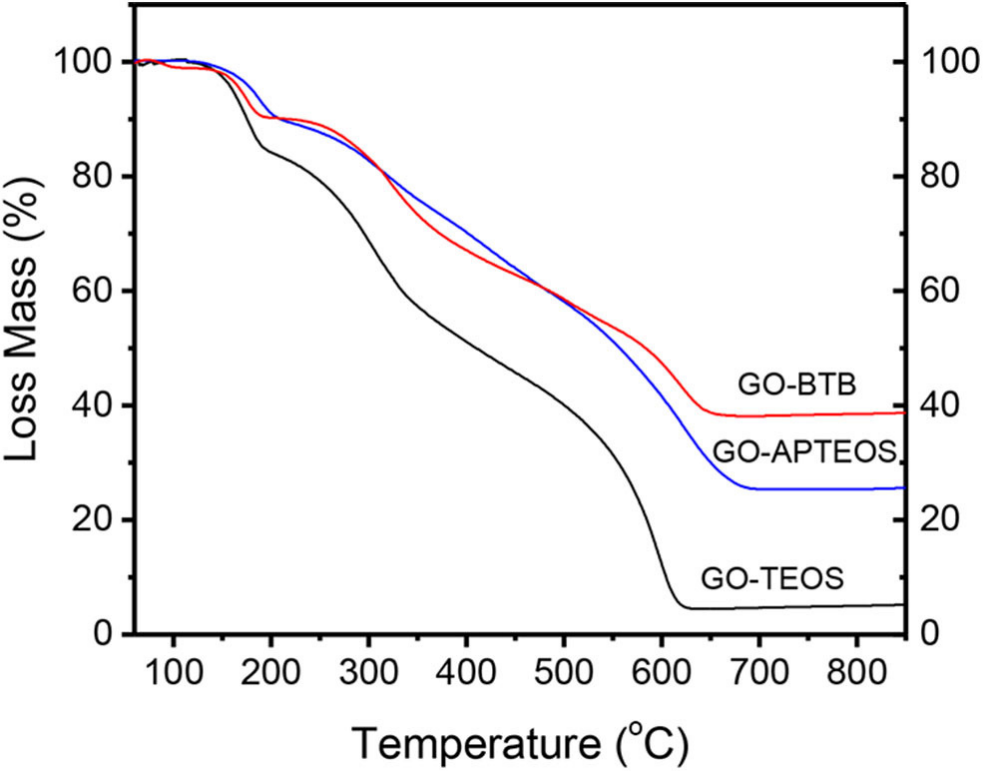
TGA curves of heterostructures prepared by silylation of organo-modified graphene oxide with 1,4-Bis(triethoxysilyl)-benzene (GO-BTB), with (3-aminopropyl)triethoxysilane (GO-APTEOS) and with tetraethylorthosilicate (GO-TEOS).

The relative amount of silicon oxide in the three porous structures can be deduced by TGA; Figure 5 presents the results for all three heterostructures. The initial, relatively small weight loss up to about 250 °C is due to the removal of the adsorbed water and of hydroxy, epoxy and carboxyl groups present in the graphene oxide layers (Bourlinos et al., 2003). When the temperature increases to 450 °C, combustion of dodecylamine takes place (Kooli et al., 2016), while in the temperature range between 500 and 700 °C, the loss mass attests to the combustion of the graphene layers (Jeong et al., 2009) as well as to the dehydroxylation of the silica networks (Ek et al., 2001). The residual mass of 8 % for GO-TEOS, 30 % for GO-APTEOS and 40 % for GO-BTB, corresponds to silicon oxide in each case. These percentages can be explained by the chemical structure of each silica precursor: for APTEOS and BTB alkoxides a larger amount of silica precursor was activated/reacted to give GO-APTEOS and GO-BTB than for the modification with TEOS, which does not have any functional group favoring bonding.

**Figure 6:**
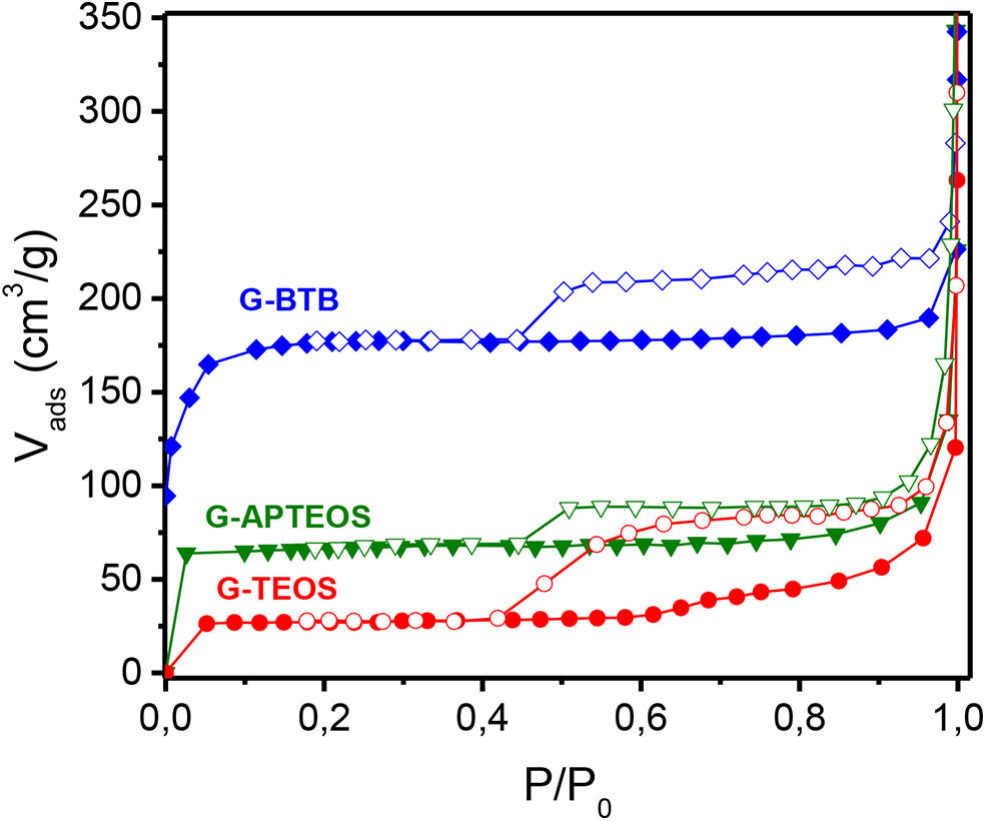
N_2_ adsorption (full symbols)-desorption (empty symbols) isotherms at 77 K of heterostructures prepared by silylation of organo-modified graphene oxide with either 1,4-Bis(triethoxysilyl)-benzene, or with (3-aminopropyl)triethoxysilane, or with tetraethylorthosilicate, and calcinated to give G-BTB, G-APTEOS and G-TEOS.

To determine the surface area of the heterostructures after calcination (G-BTB, G-APTEOS and G-TEOS), we monitored N_2_ adsorption and desorption at 77 K. Figure 6 shows the isotherms obtained, all characterized by an H4 hysteresis loop (based on IUPAC classification) (Sing et al., 1985; Kalantzopoulos et al., 2014), with a shape typical of slit-shaped pores (Letaïef et al., 2006; Qian et al., 2008). At low relative pressures (P/P_0_ < 0.01), the G-BTB heterostructure shows the highest N_2_ adsorption, which indicates that a significant amount of micropores is accessible after the creation of the silica network. For this system the BET surface area was calculated to be 576 m^2^/g, or more than twice that of G-APTEOS (227 m^2^/g) and more than 20 times that of G-TEOS (27 m^2^/g). Note that for all three heterostructures the final increase of the N_2_ uptake at relative pressures above 0.95 is attributed to adsorption on the external surface and/or the surface of macropores.

**Figure 7:**
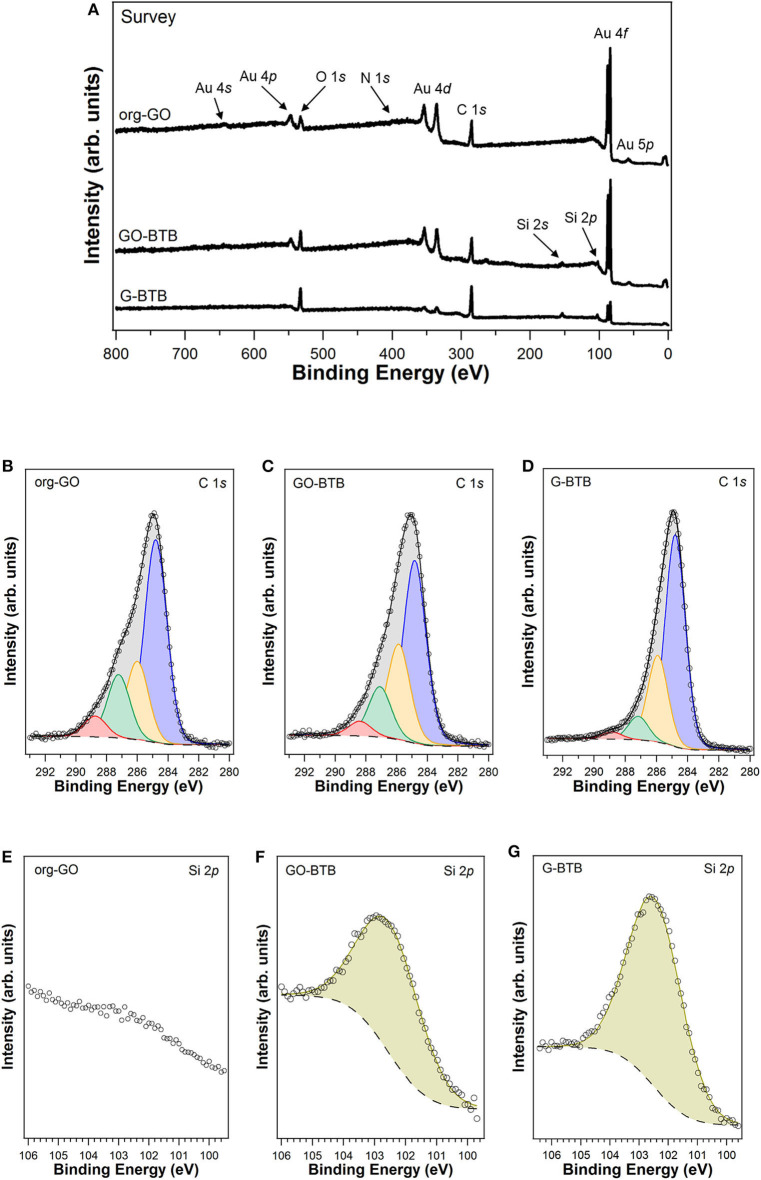
Survey scan [top panel **(A)**], C1*s* **(B–D)** and Si2*p* **(E–G)** XPS spectra of graphene oxide intercalated with dodecylamine before (org-GO), after silylation with 1,4-Bis(triethoxysilyl)-benzene (GO-BTB) and after subsequent calcination (G-BTB) (dots) and corresponding fits (full lines); for the differently colored peaks see text.

Hence, we followed all modification steps leading to the G-BTB sample by X-ray photoelectron spectroscopy. XPS survey scan of the G-BTB is shown in Figure 7A (top panel). All the expected elements are observed and there is no indication of presence of any kind of contaminant. C and O are the main constituents of the sample, while the Au signature is ascribed to the gold surface where the sample was deposited. The C:Si ratio amounts to 6.8±0.6, which is comparable with the C:Si ratio before the calcination step, and confirms the presence of BTB molecules in the GO/dodecylamine matrix.

The C1*s* spectrum of org-GO in Figure 7B shows an asymmetrical peak, which requires four components to obtain a good fit; the main component at a binding energy of 284.8 eV (marked in blue in Figure 7B) can be ascribed to C-C/C=C species, while the other peaks located at 286.0 eV, 287.2 and 288.7 eV can be assigned to C-O/C-N (yellow), C=O/C-O-C (green), COOH (light red) bonds, respectively (Spyrou et al., 2015; Stathi et al., 2015). The same four components also contribute to the C1*s* line in the cases of GO-BTB (see Figure 7C) and of G-BTB (see Figure 7D). All percentages indicating the relative amounts of carbon atoms involved in each type of bond as deduced from the XPS measurements for org-GO, GO-BTB and G-BTB are shown in Table 1. The O1*s* core levels of all the samples are shown in the Supporting Information (see Figure S2). The corresponding Si2*p* lines of GO-BTB and G-BTB in Figures 7F,G are centered at 102.5 eV, a binding energy typical of Si-O/Si-O-C species (Kaur et al., 2016). As expected no Si signal is found for org-GO (Figure 7E). In addition, the Si2*s* core level spectrum was also acquired to corroborate the presence of only one chemical species of silicon in both samples (see Figure S3 in the Supporting Information). The Si-O-C component is difficult to identify in the C1*s* peak because of the strong C-C/C=C and C-O/C-N signals (Avila et al., 2001).

**Table 1:** Binding energies and percentages indicating how much the component contributes to the total C1*s* intensity; these percentages indicate the relative amounts of carbon atoms involved in each type of bond as deduced from the XPS measurements.

**Carbon species**	**B.E. (eV)**	**Sample name and %**	**Sample name and %**	**Sample name and %**
C-Si	283.8	–	–	GO-APTEOS 5.6
C-C/C=C	284.8	org-GO 55.5 GO-BTB 53.2 G-BTB 70.4	GO-TEOS 40.7	GO-APTEOS 40.3
C-O/C-N	285.9 – 286.2	org-GO 21.5 GO-BTB 27.8	GO-TEOS 30.5	GO-APTEOS 35.4
C-O	285.9	G-BTB 21.2	–	–
C=O/C-O-C	287.1–287.4	org-GO 17.3 GO-BTB 14.7 G-BTB 6.3	GO-TEOS 21	GO-APTEOS 14
COOH	288.4–288.9	org-GO 5.7 GO-BTB 4.3 G-BTB 2.1	GO-TEOS 7.8	GO-APTEOS 4.7

**Figure 8:**
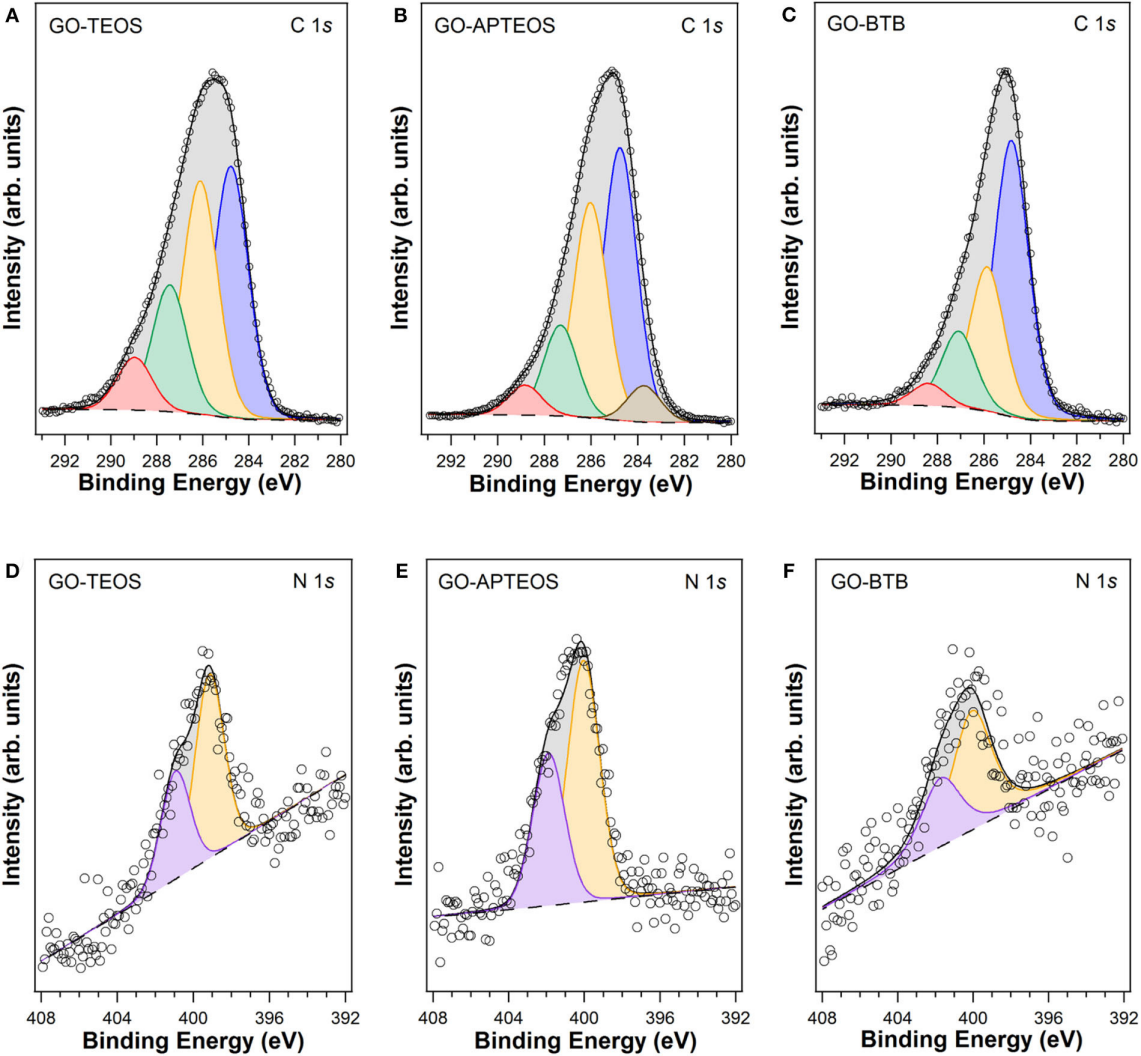
XPS spectra of the C1*s* **(A–C)** and N1*s* **(D–F)** core level regions of heterostructures prepared by silylation of organo-modified graphene oxide with 1,4-Bis(triethoxysilyl)-benzene (GO-BTB), with (3-aminopropyl)triethoxysilane (GO-APTEOS) and with tetraethylorthosilicate (GO-TEOS) (dots) and corresponding fits (full lines); for the differently colored peaks see text.

The presence of the other two precursors employed in the sol-gel synthesis, which lead to GO-TEOS and GO-APTEOS is also confirmed by X-Ray photoelectron spectroscopy. The C1*s* and N1*s* XPS spectra are depicted in Figure 8. The C1*s* photoemission spectra of GO-TEOS (Figure 8A) and GO-APTEOS (Figure 8B) are similar to GO-BTB before calcination, discussed above and shown again for comparison (Figure 8C). Table 1 summarizes the results of the fits in terms of binding energies, attribution of the various components and relative contribution to the total C1*s* intensity. Since XPS is a quantitative technique, these percentages indicate the relative amounts of carbon atoms involved in each type of bond.

The presence of nitrogen from the intercalated dodecylamine for GO-TEOS, GO-APTEOS and GO-BTB is confirmed by the N1*s* photoemission lines, shown in Figures 8D–F; note that its position in binding energy at approximately 400.0 eV (yellow in Figure 8) indicates that there is no C-N-C bond, which would give rise to a spectral signature at lower binding energies, but that the amines prefer to bind electrostatically with the oxygen groups of the graphene oxide (Cecchet et al., 2003). The peak at higher binding energies for all hybrid materials (lilac colored in Figure 8) arises from protonated amines that may interact as well with the oxygen functional groups of GO (C-OH and C(O)O). In the case of GO-APTEOS there is also the contribution from the amine end groups of APTEOS, which do not participate in the sol-gel reaction.

From the areas of C1*s* and Si2*p* lines in the XPS spectra, we calculated the C to Si ratio for all three heterostructures, taking into account the sensitivity factors for each element. GO-BTB (C:Si=6.8±0.5) contains a higher amount of Si than GO-APTEOS (C:Si=65.3±0.9), and GO-TEOS (C:Si=101.4±4.1) ranks lowest in Si content, in agreement with the TGA results discussed above.

**Figure 9:**
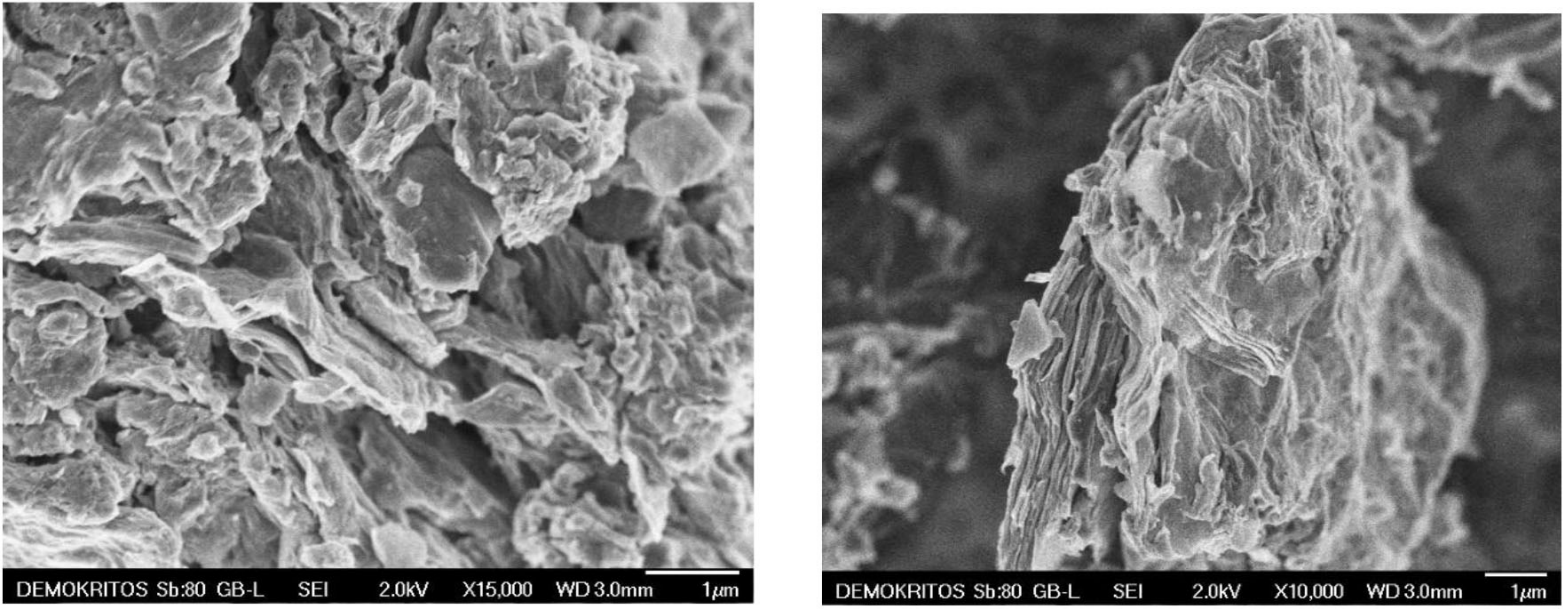
SEM images of the heterostructure prepared by silylation of organo-modified graphene oxide with 1,4-Bis(triethoxysilyl)-benzene, and calcinated to give G-BTB.

Representative scanning electron micrographs of G-BTB are shown in Figure 9. Both SEM images (a) and (b) reveal the layered nature of the porous hybrid heterostructure, confirming the high degree of exfoliation of the GO after silylation and calcination.

**Figure 10:**
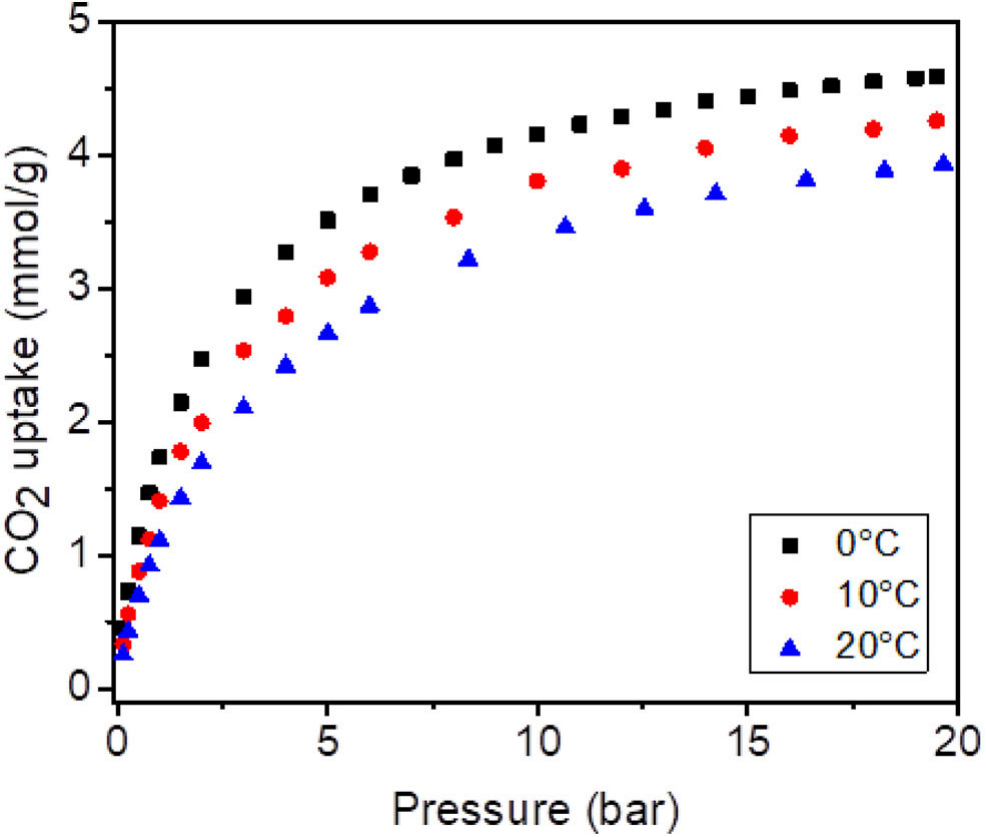
CO_2_ adsorption at 0, 10, and 20°C on the heterostructure, prepared by silylation of organo-modified graphene oxide with 1,4-Bis(triethoxysilyl)-benzene, and calcinated to give G-BTB.

The CO_2_ capture performance of G-BTB was studied at three different temperatures (0, 10, and 20 °C) and for different pressures. The results are presented in Figure 10. From these data one deduces CO_2_ adsorption capacities of 3.5 mmol/g at 5 bar and of 4.5 mmol/g at 17 bar and 0 °C. At higher temperatures the capacity is slightly lower, which is probably because the kinetic energy of the CO_2_ gas molecules is higher and hence the molecules desorb more easily (lower sticking coefficient). Such high values agree with the high BET surface area and the presence of microporosity. Furthermore, the CO_2_ adsorption capacity is comparable with that of other graphene-based porous materials with higher surface areas, measured under the same conditions (Chowdhury and Balasubramanian, 2016) This implies that there is potential for achieving even higher values with further structural optimization. Thus, G-BTB appears to be a very interesting candidate as CO_2_ storage material, which combines the properties of graphene with the very high porosity of silica resulting from the sol-gel procedure.

## Conclusions

Graphene-based porous heterostructures were produced by combining organo-modified graphene oxide with three different organo-silica precursors through sol-gel reactions. After one-step silylation, the interlayer space increased for all the heterostructures compared to organo-modified GO and was maximum for 1,4-Bis(triethoxysilyl)-benzene due to steric effects. FTIR and XPS spectroscopies gave evidence for the reduction of graphene oxide to graphene after calcination of the heterostructures and confirmed the presence of silica oxide. Thermogravimetric analysis allowed to evaluate the degree of silylation for each silica precursor, and to identify 1,4-Bis(triethoxysilyl)-benzene (with the bridged phenyl group) as the one giving the highest yield. Thermal treatment is necessary to obtain highly porous materials with sponge-like structures, characterized by a BET surface area of 550 m^2^/g in the case of G-BTB. The latter heterostructure was found to have a high CO_2_ adsorption capacity of 3.5 mmol/g at 5 bar and 0 °C, which is promising for further consideration as CO_2_ storage material.

## Data Availability Statement

The raw data supporting the conclusions of this article will be made available by the authors, without undue reservation.

## Author Contributions

AE, DG, and PR designed the experiments and finalized the manuscript. ET and ED performed the synthesis, characterization, and data analysis. EM and EMA participated in the synthesis of the materials. KS and ODL conducted the XPS measurements and analyzed the data. ET, ED, and KS wrote the manuscript. LB performed the SEM measurements. AS performed the CO_2_ measurements. All authors contributed to manuscript revision, read, and approved the submitted version.

## Conflict of Interest

The authors declare that the research was conducted in the absence of any commercial or financial relationships that could be construed as a potential conflict of interest.
