# Supplementary material for: New Porous Heterostructures Based on Organo-Modified Graphene Oxide for CO2 Capture
Source: Front Chem. 2020 Sep 17;8:564838. doi: 10.3389/fchem.2020.564838 (PMC7528310; doi:10.3389/fchem.2020.564838)
Supplement: Supplementary file 1 [file Table_1.DOCX]

Supplementary Material

**New Porous Heterostructures Based on Organo-Modified Graphene Oxide for CO_2_ capture**

Eleni Thomou^1,2†^, Evmorfia K. Diamanti^1,2†^, Apostolos Enotiadis^3*^, Konstantinos Spyrou^1^, Efstratia Mitsari^1^, Lamprini G. Boutsika^3^, Andreas Sapalidis^3^, Estela Moretón Alfonsín^2^, Oreste De Luca^2^, Dimitrios Gournis^1*^ and Petra Rudolf^2*^

^1^Department of Materials Science and Engineering, University of Ioannina, Ioannina, Greece

^2^Zernike Institute for Advanced Materials, Faculty of Science and Engineering, University of Groningen, Groningen, The Netherlands

^3^National Center for Scientific Research ‘Demokritos’, Ag. Paraskevi Attikis, Athens, Greece

*** Correspondence:**Apostolos Enotiadis
[aenotiadis@gmail.com](mailto:aenotiadis@gmail.com)

Dimitrios Gournis

[dgourni@uoi.gr](mailto:dgourni@uoi.gr)

Petra Rudolf

p.rudolf@rug.nl

1. **FTIR measurements**

**Supplementary Figure 1** FTIR spectra of graphene oxide intercalated with dodecylamine (org-GO) silylated with tetraethylorthosilicate (GO-TEOS - top panel) and (3-aminopropyl)triethoxysilane (GO-APTEOS - bottom panel) and subsequently calcinated (G-TEOS – top panel, G-APTEOS – bottom panel)

1. **X-ray photoelectron spectroscopy (XPS) measurements**

**
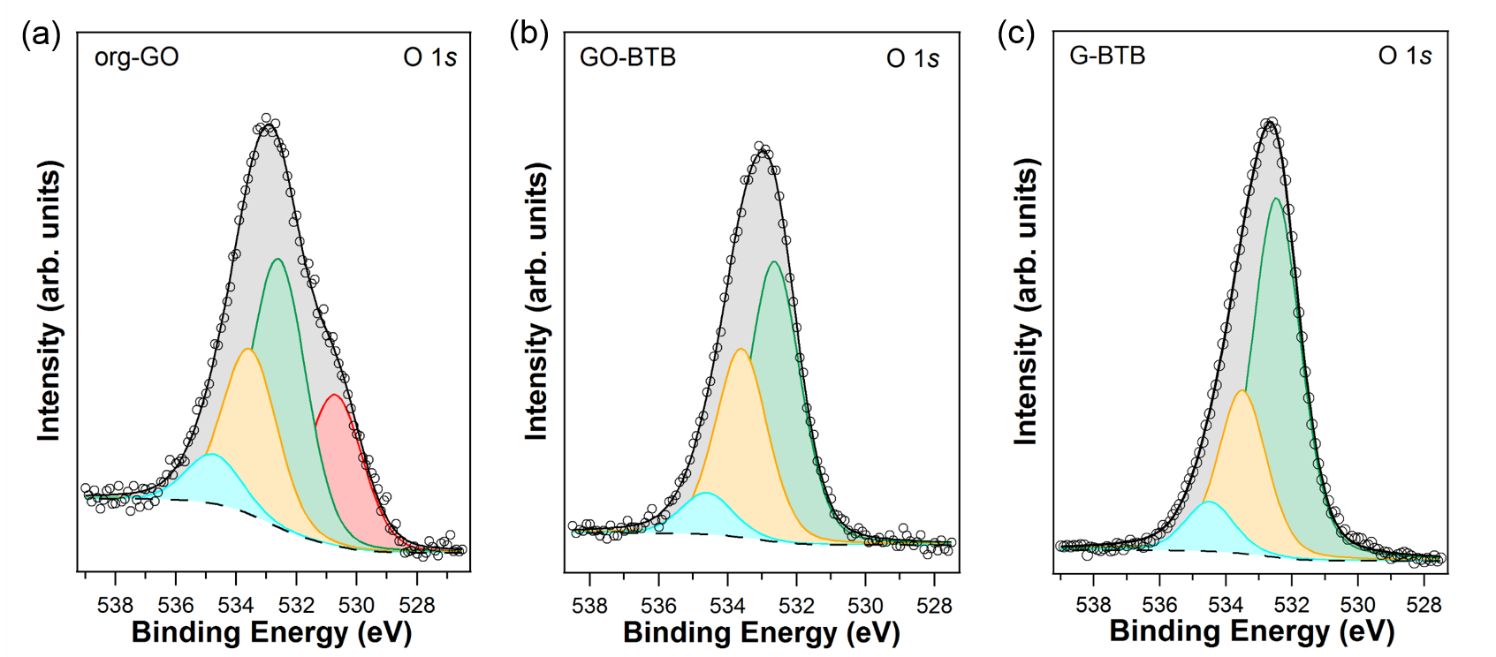
Supplementary Figure 2** XPS spectra of the O1*s* core level region of graphene oxide intercalated with dodecylamine (a) before (org-GO) and (b) after silylation with 1,4-Bis(triethoxysilyl)-benzene (GO-BTB) and (c) after subsequent calcination (G-BTB). The red component is assigned to COO chemical species while the green and yellow components are ascribed to C=O/Si-O-C/C-Si-O and C-O respectively. Finally, the light blue peak is attributed to adsorbed water (Haubner *et al.*, 2010).

**
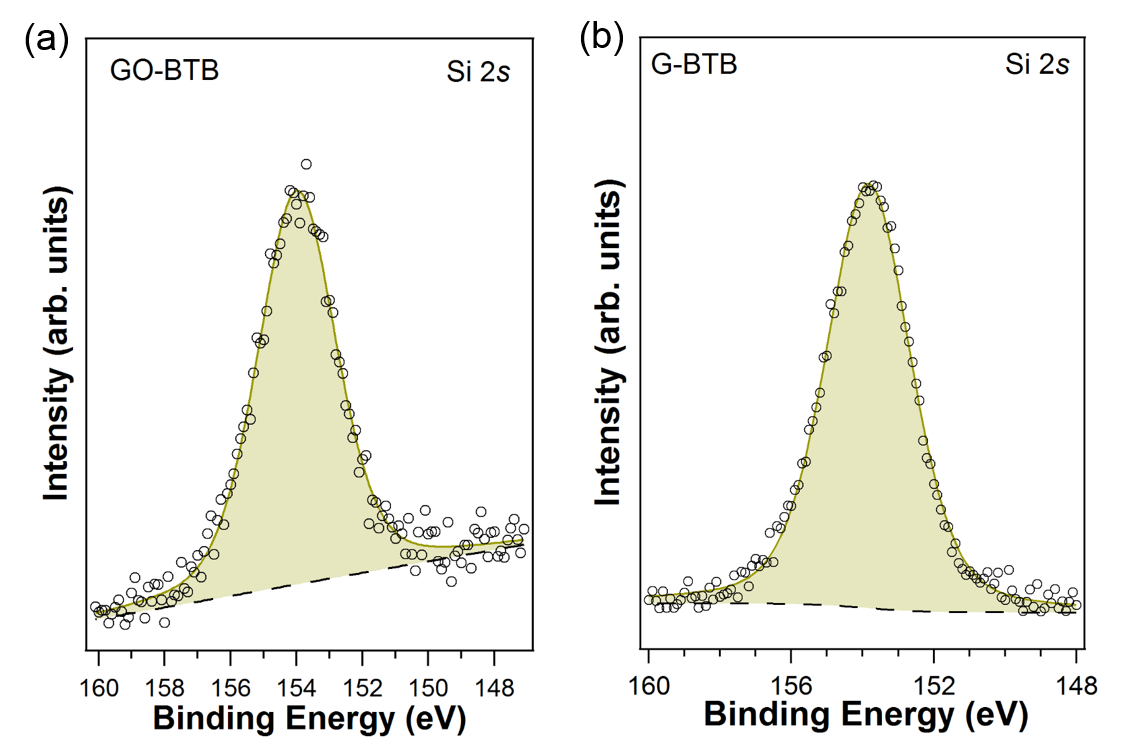
**

**Supplementary Figure 3** XPS spectra of the Si2*s* core level region of (a) GO-BTB and (b) G-BTB samples.

**References**

Haubner, K., Murawski, J., Olk, P., Eng, L. M., Ziegler, C., Adolphi, B., & Jaehne, E. (2010). The route to functional graphene oxide. *ChemPhysChem*, 11(10), 2131-2139
